# Nonhealing Ulcer: Acroangiodermatitis of Mali

**DOI:** 10.1155/2011/909383

**Published:** 2011-10-25

**Authors:** Neeraj Varyani, Anubhav Thukral, Nilesh Kumar, Kailash Kumar Gupta, Ravi Tandon, Kamlakar Tripathi

**Affiliations:** Department of General Medicine, Institute of Medical Sciences, Banaras Hindu University, Uttar Pradesh, Varanasi 221005, India

## Abstract

An 18-year-old male presented with a nonhealing wound on left lower limb, pain and swelling over multiple joints, weight loss, and yellowish discoloration of eyes and urine for the past 4 years. On examination, the patient had pallor, icterus, and generalized lymphadenopathy with a nonhealing unhealthy ulcer over left medial malleolus. He had deformed joints with hepatomegaly and splenomegaly. His laboratory investigations were positive for antinuclear antibody (ANA) and anticardiolipin antibody (ACLA). Synovial fluid analysis showed inflammatory findings. Biopsy of margin of the ulcer showed findings consistent with *Acroangiodermatitis of Mali*. The patient was treated with disease-modifying antirheumatic drugs (DMARDs) and aspirin for juvenile idiopathic arthritis and secondary antiphospholipid antibody syndrome (APS), respectively. The ulcer was managed conservatively with systemic antibiotics and topical steroids along with limb elevation and compression elastic stockings. The patient's symptoms improved significantly, and he is in our followup.

## 1. Introduction

Nonhealing ulcer is a cause for significant morbidity and mortality. Such an ulcer must undergo a biopsy so that an appropriate diagnosis can be reached and treatment instituted rapidly Acroangiodermatitis of Mali is an uncommon but not rare cause of nonhealing wound in the lower limbs. Only 100 cases have been reported so far in the USA, a major cause being underdiagnosing and underreporting of this condition. Even after extensive search in Cochrane library and Pubmed, we were not able to find a case report with juvenile idiopathic arthritis and acroangiodermatitis of Mali which makes our case even more important. Its differentiation from Kaposi's sarcoma is very essential and requires biopsy for conformation.

## 2. Case Presentation

On August 2010, an 18-year-old male presented to our outpatient department, complaining of chronic non healing leg wound on left lower limb for 4 years, pain and swelling of bilateral wrist, elbow and knee joint with morning stiffness for 8 months, weight loss and yellowish discoloration of eyes and urine for 3 months, and intermittent fever for last 1 month. The wound was painful, nonitchy, just over the medial malleolus of left leg, had well-defined indurated margins, surrounding hyperpigmentation, with no discharge or bleeding from its surface. There was no history of rash, oral ulceration, photosensitivity, or bleeding from any other site. He had a history of blood transfusions 2 years back and a similar non healing ulcer on the right leg which persisted for about 6 months to eventually heal with repeated dressings.

On examination our patient had severe pallor, icterus, cachexia, Marfanoid habitus, generalized lymphadenopathy with stable vitals but no thyromegaly. All the peripheral pulses were palpable. A large ulcer over medial malleolus of left leg was noted with lipodermatosclerotic changes of the surrounding skin. Swelling over bilateral wrist, elbow, and knee joints were present with flexion deformity at left knee joint. He had a painful gait due to the deformities. Abdominal examination revealed splenomegaly and hepatomegaly. On nervous system examination there was no sensory or motor deficit or any peripheral nerve thickening or tenderness. Fundus and other systemic examinations were normal. Hematological investigations revealed anemia (50 gm/l), total bilirubin (78.66 mcmol/l), indirect bilirubin (64.98 mcmol/l), total serum protein (82 g/l), and serum albumin (35 g/l). He had ANA, ACLA (36.0 arbitrary units), and lupus anticoagulant tests positive at time of presentation and when repeated 12 weeks later. IgM rheumatoid factor was negative (4.7 KIU/L). C-reactive protein (CRP) test was positive (44.6 mg/L). Enzyme-Linked immunosorbent assay (ELISA) test for human immunodeficiency virus (HIV), surface antigen for hepatitis B, and IgM antibody to hepatitis C were also negative. General blood picture was unremarkable. Reticulocyte count was 0.4%. Direct and indirect Coombs test and the test for sickling were negative. Hemoglobin electrophoresis suggested beta-thalassemia trait. Venereal disease research laboratory (VDRL) test was nonreactive. Fine needle aspiration cytology (FNAC) of axillary lymph node showed reactive lymphoid hyperplasia. Radiograph of bilateral wrist and hand joint showed juxta-articular osteopenia without any deformity. Radiograph of sacroiliac joint showed bilateral sacroiliitis. Synovial fluid analysis revealed yellow-colored, turbid, viscous fluid with 5318 cells/microlitre, PMNs forming 90% of it; its culture was negative suggesting inflammatory joint swelling.

**Figure 1:**
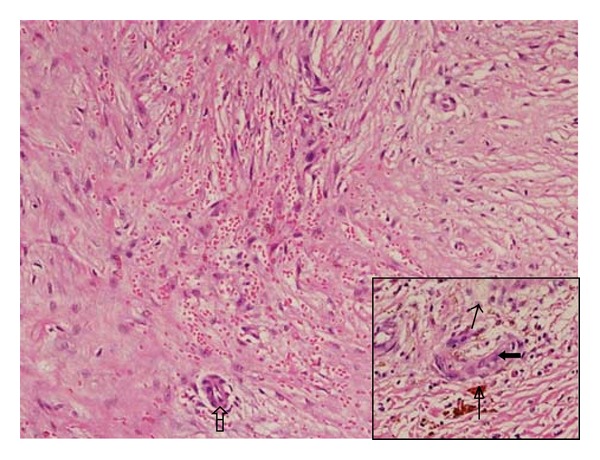
400x; histopathology showing thick-walled capillaries (closed arrow in inset and open arrow in main figure), hemosiderin deposition with golden brown color (arrow in inset), and sparse perivascular lymphocytic infiltrate (thin arrow in inset).

**Figure 2:**
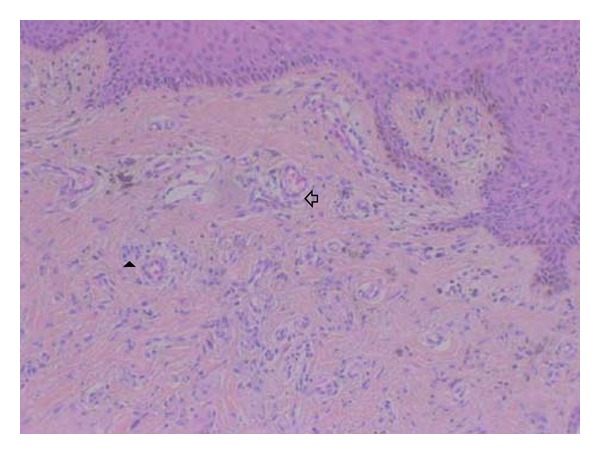
100x; histopathology showing perivascular lymphocytic infiltrate (arrow head) and thick-walled capillaries (hollow arrow).

Based on the clinical profile of the patient an initial diagnosis of venous ulcer and a differential diagnosis of vasculitic ulcer were made. However, color Doppler of bilateral lower limbs and abdomen was normal with no evidence of arteriovenous fistula or venous insufficiency. Biopsy from the edge of the ulcer revealed increased number of thick-walled capillaries that were present in a clustered pattern within papillary dermis (Figures 1 and 2). Sparse perivascular lymphocytic infiltrate (Figures 1 and 2) and hemosiderin deposition (Figure 2, insight) were also present, and diagnosis of acroangiodermatitis of Mali was made.

 We managed our patient aggressively with methotrexate at a dose of 10 mg weekly, corticosteroids (prednisolone) at 1 mg/kg/day, leflunomide at a dose of 20 mg daily, aspirin at 75 mg/day, and with repeated aseptic dressings of the ulcer and topical steroid application along with compression stockings and limb elevation during night hours. The patient is in our followup and has shown improvement.

## 3. Discussion

Acroangiodermatitis (pseudo-Kaposi sarcoma) is a chronic dermatosis and is associated with venous insufficiency or with vascular anomalies of any cause such as Klippel-Trenaunay syndrome as discussed by Lyle and Given [1] or as stump dermatosis in amputees as shown in the work by Badell et al. [2]. It has been reported in patients with a thrombophilic prothrombin mutation as discussed by Martin et al. [3], in upper limbs following placement of arteriovenous shunt for hemodialysis as shown by the extensive work by Fernández et al. [4], in the paralyzed limb as shown by the study of Landthaler et al. [5], and hepatitis C. It is more prevalent in males. Etiopathogenesis continues to be an enigma. Acroangiodermatitis arises from the hyperplasia of preexisting blood vessels as opposed to Kaposi sarcoma in which vascular proliferation is independent of the existing vessels. It is usually a complication of chronic venous stasis and venous hypertension of lower limbs. Severe chronic venous stasis and the insufficiency of the calf muscle pump result in an elevated capillary pressure. In paralyzed limbs, lack of muscle pump, disturbed innervations of vessels, venous stasis, and enhancing arteriovenous channels have been suggested to be involved in its pathogenesis. Lesions occur especially on lower legs but may extend on to the dorsum of the feet and toes, and even up the leg, especially over dilated varicosities. Individual lesions are minute purpuric macules that coalesce to form irregular plaques, which may be several centimeters in diameter. Color of the lesion varies from purple to yellow to brown depending upon the amount of hemosiderin deposition. Epidermis may be normal or show mild eczematous changes. Its differential diagnoses are Kaposi sarcoma, gravitational dermatitis, and Schamberg's disease; the former which is the most important differential can be distinguished by staining pattern with CD34 antigen, which stains both endothelial cells and perivascular spindle cells in Kaposi's sarcoma but only the endothelial cells in acroangiodermatitis as shown by Kanitakis et al. [6]. Absence of HHV-8 expression in acroangiodermatitis of Mali also helps in its differentiation from Kaposi sarcoma [4]. On histopathology, papules and nodules consist of a proliferation of small dilated vessels in an edematous dermis. The vessels have fairly regular profiles and lack the jagged outline characteristic of Kaposi sarcoma as discussed by Gottlieb and Ackerman [7]. Plump endothelial cells without atypia line the vessels. The cells are positive for CD34 [3]. A slight perivascular fibroblastic proliferation is also seen but is not marked. Some lesions show nodular collections of vessels with narrow lumina as shown by Strutton and Weedon [8]. Extravasated red blood cells, hemosiderin, and a variable round-cell infiltrate are seen around the vascular proliferation. Plasma cells are usually not present. The overlying epithelium may show hyperkeratosis. Treatment of pseudo-Kaposi sarcoma is unsatisfactory and disappointing, but pressure bandage seems logical. Many dermatologists have tried systemic antibiotics and topical steroids with varying results.

This case emphasizes that a non-healing ulcer should always undergo biopsy, and appropriate diagnosis can help improve the quality of life of patients immensely. Examination of the skin which is often neglected in internal medicine should be routine initial part of examination of every patient.
